# Tumor Immunotherapy Using A_2A_ Adenosine Receptor Antagonists

**DOI:** 10.3390/ph13090237

**Published:** 2020-09-08

**Authors:** Jinfeng Zhang, Wenzhong Yan, Wenwen Duan, Kurt Wüthrich, Jianjun Cheng

**Affiliations:** 1 iHuman Institute, ShanghaiTech University, Shanghai 201210, China; zhangjf@shanghaitech.edu.cn (J.Z.); yanwzh@shanghaitech.edu.cn (W.Y.); duanww@shanghaitech.edu.cn (W.D.); kw@mol.biol.ethz.ch (K.W.); 2 School of Life Science and Technology, ShanghaiTech University, Shanghai 201210, China; 3 Department of Integrated Structural and Computational Biology, Scripps Research, La Jolla, CA 92037, USA

**Keywords:** GPCR, immuno-oncology, Parkinson’s disease, drug binding modes, cancer therapy

## Abstract

The A_2A_ adenosine receptor (A_2A_AR) plays critical roles in human physiology and pathophysiology, which makes it an important drug target. Previous drug-discovery efforts targeting the A_2A_AR have been focused on the use of A_2A_AR antagonists for the treatment of Parkinson’s disease. More recently, the A_2A_AR has attracted additional attention for its roles in immuno-oncology, and a number of A_2A_AR antagonists are currently used as lead compounds for antitumor drugs in both preclinical models and clinical trials. This review surveys recent advances in the development of A_2A_AR antagonists for cancer immunotherapy. The therapeutic potential of representative A_2A_AR antagonists is discussed based on both animal efficacy studies and clinical data.

## 1. Introduction

The A_2A_ adenosine receptor (A_2A_AR) is a family A G protein-coupled receptor (GPCR) [1]. There are four subtypes of adenosine receptors (A_1_, A_2A_, A_2B_, and A_3_), and all of them can be activated by extracellular adenosine [1]. The A_1_ and A_2A_ receptors are widely distributed in both the central nervous system (CNS) and the periphery, while the density of A_2B_ and A_3_ in the brain is very low [2]. The A_1_, A_2A_, and A_2B_ adenosine receptors are conserved throughout evolution and are highly homologous across different species, whereas A_3_ varies substantially [3]. Sequence homology is about 49% between A_1_ and A_3_, and 59% between the A_2A_ and A_2B_ receptors. In terms of endogenous ligand binding, the A_1_, A_2A_, and A_3_ receptors have high affinity, whereas A_2B_ shows low binding affinity for adenosine [4]. Upon adenosine binding and the resulting activation, the A_1_ and A_3_ receptors couple primarily to the G_i_ protein, leading to a decrease in the intracellular concentration of cAMP, whereas A_2A_ and A_2B_ couple preferentially to the G_s_ protein, which leads to an increase in intracellular cAMP levels [4]. These diverse downstream effects of the different adenosine receptor subtypes require high selectivity of synthetic ligands to be used as A_2A_AR antagonists [5].

Over the past decades, drug-discovery efforts have led to numerous A_2A_AR-targeting ligands, including both agonists and antagonists [6]. A_2A_AR agonists have been examined as anti-inflammatory agents and as coronary vasodilators [7]. Regadenoson, a selective A_2A_AR agonist, has been approved as a pharmacological stress agent in myocardial perfusion imaging (MPI) [8]. Early studies of A_2A_AR antagonists have been focused on their use to treat Parkinson’s disease (PD) [9,10]. This is based on the fact that A_2A_AR is prone to forming heterodimers with the dopamine D2 receptor in the CNS, and that activation of A_2A_AR inhibits the activation of the D2 receptor [2]. A_2A_AR antagonists can prevent this inhibition and enhance dopaminergic activity, leading to the desired therapeutic effects in PD patients. A number of A_2A_AR antagonists have shown encouraging anti-Parkinson effects in animal models of PD, and a number of clinical trials have been conducted to evaluate their therapeutic potential [11]. These include the compounds istradefylline (also known as KW-6002, Kyowa Hakko Kirin), PBF-509 (PaloBiofarma), V81444 (Vernalis), ST1535 and ST4206 (Sigma-Tau), V2006 (Biogen), SCH-420814 (Merck Sharp & Dohme), and SYN115 (Biotie Therapies) [9]. Although most of these compounds failed to show statistically significant therapeutic effects in the clinic, istradefylline showed efficacy as an adjunctive treatment to levodopa/carbidopa in PD patients by reducing the “off” episodes. It was approved in Japan in 2013 [12] and was also approved by the US FDA in August 2019.

The role of adenosine as an immunosuppressive factor was first reported in 1975, when it was demonstrated that adenosine inhibits lymphocyte-mediated cytolysis via increasing intracellular cAMP levels [13]. It was later reported that the concentration of adenosine is significantly enhanced compared to adjacent tissues in the tumor microenvironment (TME) [14], creating an “adenosine protective ring” that helps the tumor to fight off attacks from the immune system. Regarding the immunosuppressive role of adenosine, it has been demonstrated that among the four adenosine receptor subtypes, A_2A_AR is the dominant receptor for extracellular adenosine, leading to an increase in intracellular cAMP and consequently the functional inhibition of immune cells. Genetic knockout of the A_2A_AR suppressed the immunosuppression by adenosine, and small molecule A_2A_AR antagonists have similar effects [15,16]. Overall, studies with different A_2A_AR antagonists have thus shown that A_2A_AR is a promising target for the development of novel immunotherapies of cancer [17,18,19].

When A_2A_AR gained interest as an immuno-oncology drug target, a small library of A_2A_AR antagonists had already been reported, mainly as a result of earlier efforts in the development of A_2A_AR antagonists as anti-PD agents. This greatly facilitated the mechanism-of-action and proof-of-concept studies of A_2A_AR antagonists as novel anticancer agents. Furthermore, the A_2A_AR is one of the most extensively studied GPCRs in structural biology, being one of the GPCRs for which both the antagonist-bound and agonist-bound structures have been reported [20,21]. The structure of an agonist-A_2A_AR-G_s_ ternary complex has also been reported [22]. Conformational dynamics of the A_2A_AR have been demonstrated recently, using solution NMR methods [23,24]. These structural biology data greatly facilitate the design of novel compounds with high binding affinity and target selectivity. The promising role of A_2A_AR in immuno-oncology and the potential of combination therapies using A_2A_AR antagonists together with other checkpoint inhibitors, encourage continuous efforts in this area. Therefore, a new wave of drug-discovery efforts targeting the A_2A_AR has been emerging.

## 2. Mechanism of Action of A_2A_AR Antagonists in Immuno-Oncology

**Figure 1:**
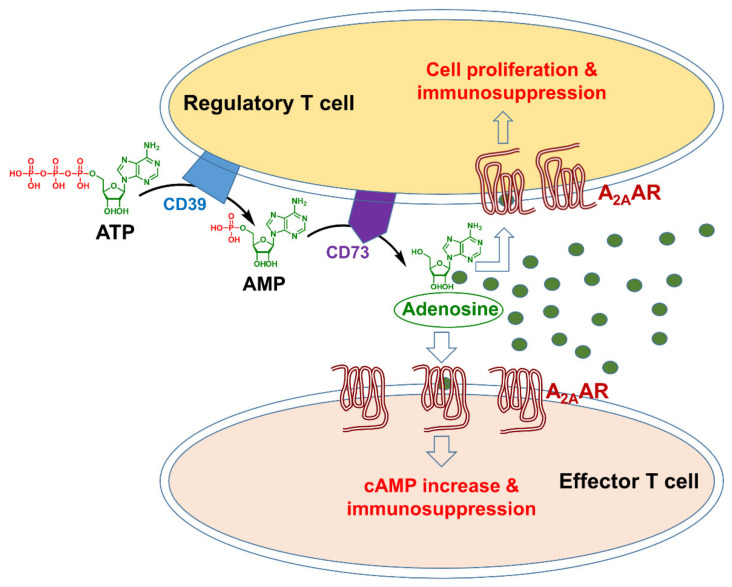
Adenosine-A_2A_AR signaling in the tumor microenvironment (the green circles represent adenosine molecules). ATP = adenosine triphosphate; AMP = adenosine monophosphate.

A line of evidence has highlighted the importance of adenosine as a critical immunosuppressive factor that accumulates in the TME [4,14,25]. The concentration of this nucleoside, which is present at low nanomolar levels in the interstitial fluids of unstressed tissues, can rapidly increase in response to pathophysiological conditions, such as hypoxia, ischemia, inflammation, or tissue injury [25]. Many factors can lead to extracellular aggregation of adenosine triphosphate (ATP) in the TME, for example, hypoxia, increased metabolism, and apoptosis. Two nucleotidases expressed on the cell surface, CD39 and CD73, catalyze the conversion of ATP to AMP (adenosine monophosphate) and of AMP to adenosine, respectively (Figure 1). CD39 is expressed by regulatory T and B cells [26], and CD73 is expressed on the regulatory T (Treg) cells and various stromal cells in the bone marrow, such as mesenchymal stem cells, fibroblasts, and endothelial cells [27]. Preclinical studies have shown that with the impact of these agents, adenosine concentrations in the TME can increase more than 10-fold [14], leading to immunosuppression by tumor tissues.

Besides its roles in the regulation of the immune system, adenosine is also involved in angiogenesis and tumor cell proliferation [4]. Different tumor types actually show altered purine metabolism, which facilitates the production of adenosine and/or reduces its degradation, thereby resulting in a “protective adenosine halo” that contributes to cancer progression [18,28].

It was reported almost two decades ago that A_2A_AR is the dominant receptor for adenosine in its immunosuppressive roles, so that blocking A_2A_AR signaling could downscale the immunosuppression by adenosine [15]. It has also been reported that A_2A_AR protects tumors from the cytotoxic effects of functional T cells [16]. Hypoxia-driven accumulation of extracellular adenosine in the TME triggers the suppression of activated immune cells via A_2A_ARs on their cell surfaces. This molecular pathway is of critical importance for immunosuppression in the TME [29]. The main immune cells that A_2A_AR acts on are CD8^+^ T cells and the natural killer (NK) cells. Blocking A_2A_AR with the antagonist SCH-58261 enhanced interferon gamma levels and the cytotoxic CD8^+^ T cell response, leading to tumor suppression [30]. The stimulation of the A_2A_AR also suppresses the maturation of NK cells and their cytotoxic effects in vitro and promoted tumor metastasis in mice by decreasing the expression of the cytotoxic protein granzyme B by NK cells [30].

Adenosine binding to A_2A_AR also suppresses antitumor immunity through its action on the Treg cells, which play a key role in regulating or suppressing the function of effector T cells in the immune system (Figure 1). In the TME, suppressed immunity by Treg cells supports the uncontrolled growth of cancer cells. An increased number of Treg cells in the TME thus represents a barrier to successful immunotherapies [31], therefore blocking the A_2A_AR prevents immunosuppression by tissue-produced adenosine as well as by Treg cells.

Adenosine also promotes angiogenesis of solid tumors [4]. Although A_2B_AR has been shown to be the main receptor that mediates the proangiogenic role of adenosine, through inducing the secretion of the vascular endothelial growth factor (VEGF) [32], A_2A_AR has also been demonstrated to play a role [4]. In the early stages of human lung cancer, the expression of A_2A_AR on endothelial cells is increased, which may indicate that these receptors are involved in promoting the growth of blood vessels and thereby support early tumor growth and proliferation [33]. Alternatively, A_2A_AR knockout led to reduced tumor angiogenesis and concomitant tumor growth suppression [34].

Many preclinical studies have shown that A_2A_AR antagonists can block the immunosuppressive effects of adenosine, making A_2A_AR antagonists a novel class of potential therapeutic agents in immuno-oncology [35]. For example, both gene knockout of A_2A_AR and its inactivation by small molecule A_2A_AR antagonists could liberate tumor-reactive CD8^+^ T cells from tumor-induced immunosuppression [34]. The combination of A_2A_AR antagonists with immune checkpoint inhibitors, such as anti-PD-1/PD-L1 or CTLA4 antibodies, led to significantly enhanced antitumor effects [19,36]. In mouse experiments, the combination of A_2A_AR antagonists with adoptive cellular immunotherapies (ACT) also showed enhanced antitumor effects [37].

Overall, the available literature emphasizes the complexity of tumor immunity, and understanding of the roles of adenosine-A_2A_AR signaling in immuno-oncology is still evolving. The important roles of A_2B_AR in the immunosuppressive roles of adenosine are worth noting [38], and simultaneous inhibition of A_2A_AR, A_2B_AR and the upstream nucleotidases CD39 and CD73 may be necessary for optimal therapeutic efficacy [4]. Preclinical studies have convincingly demonstrated the anticancer efficacy of A_2A_AR antagonists [18,30,39,40,41]. A_2A_AR antagonists have also been combined with other approaches of immunotherapy to potentiate additive effects on tumor control and markedly enhance antitumor immunity in mouse models [19,36,40,42,43]. Considering the promise of the work surveyed in this review, we can look forward to more and more A_2A_AR antagonists emerging as potential drugs in the field of immuno-oncology.

## 3. A_2A_AR Antagonists in Preclinical and Clinical Studies

**Figure 2:**
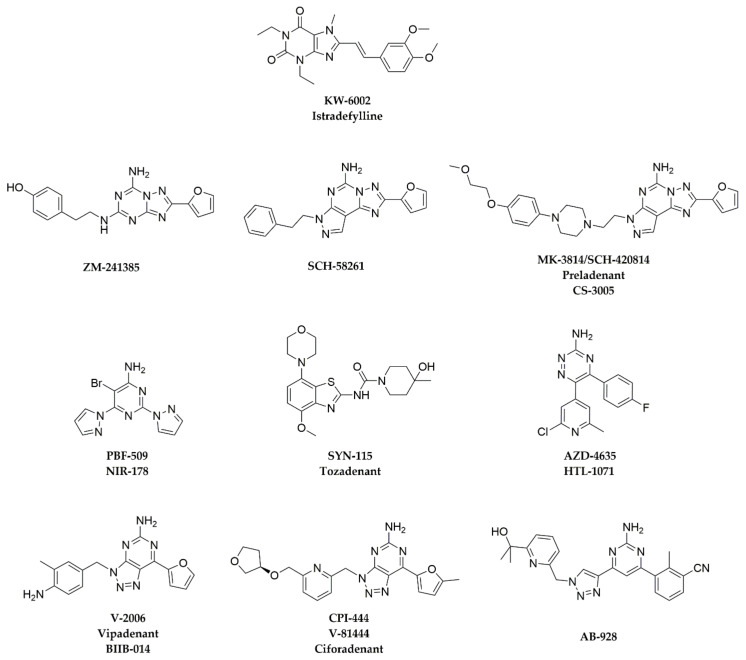
Chemical structures of A_2A_AR antagonists discussed in this chapter.

Representative A_2A_AR antagonists are reviewed in this section for which preclinical antitumor effects have been reported and/or clinical trials have been initiated. Chemical structures of these A_2A_AR antagonists are shown in Figure 2, and their binding affinities to A_2A_AR and other adenosine receptors are summarized in Table 1.

### 3.1. ZM-241385

The compound ZM-241385 was reported by Zeneca Pharmaceuticals (now AstraZeneca) as one of the first A_2A_AR antagonists. It shows a binding affinity of 0.8 nM for A_2A_AR, with 318-, 62-, and >1000-fold selectivity against A_1_AR, A_2B_AR, and A_3_AR, respectively (Table 1) [45,46]. In functional assays, it showed an IC_50_ of 54 nM in a G protein-mediated cAMP assay [52]. ZM-241385 has been widely used in biological studies of A_2A_AR [53,54], but there have been few in vivo studies in tumor models. ZM-241385 was tested both alone and in combination with an anti-CTLA4 mAb in a B16F10 mouse melanoma model, and it was found that melanoma-bearing mice treated with ZM-241365 alone showed a marked tumor growth inhibition compared with controls, and the combination therapy showed significant tumor growth delay compared with either controls or each agent alone [55]. The results showed that when an A_2A_AR antagonist is combined with an anti-CTLA4 mAb, the therapeutic effects of inhibiting tumor growth and antitumor immune responses can be enhanced [55]. However, ZM-241385 has poor pharmacokinetic properties and low bioavailability [56]. Therefore, no clinical trials have been initiated. The bicyclic non-xanthine scaffold of ZM-241385, with the furan and –NH_2_ substitutions, has served as a pharmacophore for the design of novel A_2A_AR antagonists having high selectivity (Figure 2).

### 3.2. SCH-58261

Compound SCH-58261 is a potent and selective A_2A_AR antagonist developed by Schering-Plough. It shows a binding affinity of 0.6 nM for A_2A_AR, with 478-, 8351-, and >10,000-fold selectivity against A_1A_R, A_2B_AR, and A_3_AR, respectively (Table 1) [47]. In functional assays, it showed an IC_50_ of 17 nM in the G protein-mediated cAMP assay [57]. SCH-58261 has also been widely used for characterizing A_2A_AR [58]. In CD73^+^ mouse tumor models, SCH-58261 was found to enhance tumor immunotherapy and suppress metastases [30,43]. In melanoma and breast cancer mouse models, SCH-58261 prolonged survival and reduced metastatic burden when used in combination with an anti-mouse CD73 mAb [59]. However, SCH-58261 has poor physicochemical and pharmacokinetic properties [58], and no clinical studies have been initiated.

### 3.3. MK-3814

MK-3814 (or SCH-420814, preladenant, CS-3005) is a structural derivative of SCH-58261. To improve A_2A_AR selectivity and physicochemical and pharmacokinetic properties, hydrophilic groups were added to the side chain of SCH-58261 to give SCH-420814/MK-3814 [48]. MK-3814 exhibits higher affinity for human A_2A_AR, with a K_i_ value of 1.1 nM, and high selectivity of over 1000-fold for human A_2A_AR compared with the other adenosine receptors (Table 1) [48]. MK-3814 is orally active and shows good pharmacokinetic properties [60]. A phase Ib/II study of MK-3814 used alone and in combination with the anti-PD-1 drug pembrolizumab (Keytruda) in patients with advanced solid tumors was initiated in June 2017 (NCT03099161). Unfortunately, the trial was terminated early because the data did not support the study endpoints.

### 3.4. PBF-509

PBF-509 (or NIR-178) is a potent and selective A_2A_AR antagonist discovered by Palobiofarma, which was licensed to Novartis through an agreement in 2015. It has a high affinity for A_2A_AR (K_i_ = 12 nM), with 208-, 83-, and 416-fold selectivity against A_1A_R, A_2B_AR, and A_3_AR, respectively (Table 1) [18]. It showed a K_B_ value of 72.8 nM in the agonist-mediated cAMP accumulation assay in A_2A_AR^SNAP^-expressing HEK cells [61]. PBF-509 has been tested in a mouse model of B16F10 melanoma, which was gene-modified to express CD73, as well as an MCA205 model, which endogenously expresses high levels of CD73. Oral administration of PBF-509 (15 or 30 mg/kg/day) significantly reduced the tumor burden of mice in both models [18]. A phase I/II study of PBF-509 both alone and in combination with anti-PD-1 mAb PDR001 in non-small cell lung cancer patients was started in October 2015 (NCT02403193); the results showed that the compound was well tolerated, and clinical benefit was observed in immunotherapy-exposed and -naive patients, irrespective of the PD-L1 status [62].

### 3.5. SYN-115

Compound SYN-115 (tozadenant) was first reported by Roche [49], which was later licensed to Synosia Therapeutics. SYN-115 has a benzothiophene scaffold that is structurally not related to xanthine or adenine, and it shows a binding affinity of 5 nM for A_2A_AR, with 270-, 140-, and 314-fold selectivity against A_1_AR, A_2B_AR, and A_3_AR, respectively (Table 1) [49]. SYN-115 was generally well-tolerated in phase II trials for PD and was advanced to phase III [63], but eventually the clinical trials for PD were discontinued. In a mouse model of CD73-expressing AT-3ova^dim^ breast carcinoma, SYN-115 had no single-agent activity but significantly enhanced the antitumor efficacy of the anti-PD-1 mAb RMP1-14 by promoting antitumor T-cell responses [36]. No subsequent clinical cancer studies have been registered as yet.

### 3.6. AZD-4635

AZD-4635 (HTL-1071) is an orally available A_2A_AR antagonist. It was discovered by Heptares Therapeutics (now a wholly owned subsidiary of Sosei Heptares), and AstraZeneca licensed exclusive global rights to the compound in 2015. AZD-4635 binds to human A_2A_AR with a K_i_ of 1.7 nM and with 94-, 37-, and >5000-fold selectivity against A_1_AR, A_2B_AR, and A_3_AR, respectively (Table 1) [50]. AZD-4635 was reported to inhibit adenosine-mediated cAMP accumulation in both human and mouse A_2A_AR-expressing cells [50]. For the human A_2A_AR, it inhibited the cAMP increase induced by 1 μM adenosine with an IC_50_ of 10 nM [50]. In an MC-38 syngeneic mouse colorectal tumor model, treatment with AZD-4635 at 50 mg/kg BID led to a reduction in tumor growth [50]. When used in combination with an anti-PD-L1 mAb, the tumor suppressive effects were further enhanced. AZD-4635 increased expression of the genes associated with immune activation and increased expression of co-stimulatory markers on antigen-presenting cells (APCs) [50].

AZD-4635 is currently in phase II clinical trials for the treatment of solid tumors. In June 2016, phase I studies of continuous oral monotherapy with AZD-4635 alone or in combination with various other drugs (durvalumab, abiraterone acetate, enzalutamide, oleclumab, and docetaxel) were initiated in patients with advanced solid malignancies [NCT02740985]. In May 2018, MedImmune LLC. started a clinical study of AZD-4635 in combination with the anti-CD73 antibody oleclumab (MEDI9447) to investigate the safety, tolerability, and antitumor activity of novel combination therapies administered in subjects with advanced non-small cell lung cancer (NSCLC) [NCT03381274]. In August 2019, a phase II study of AZD-4635 in patients with prostate cancer in combination with the anti-PD-L1 antibody durvalumab and anti-CD73 oleclumab was also initiated [NCT04089553]. One now looks forward to seeing if these studies will demonstrate efficacy of AZD-4635 either as a single agent and/or in combination with other anticancer therapeutics.

### 3.7. CPI-444

CPI-444 (ciforadenant, previously known as V-81444) is an orally available A_2A_AR antagonist created by Vernalis [64]. CPI-444 was designed to address the chemical structural liabilities that may have led to toxicity concerns for compound V-2006 (vipadenant, previously known as BIIB014, a non-xanthine, selective A_2A_AR antagonist [51]). It showed a binding affinity of 3.54 nM for A_2A_AR, with 54-, 431-, and 693-fold selectivity against A_1_AR, A_2B_AR, and A_3_AR, respectively (Table 1) [19]. In functional assays, CPI-444 showed an IC_50_ of 70 nM in the G protein-mediated cAMP assay [19]. In multiple murine tumor models, including the MC-38 and CT-26 colon tumors, the B16F10 melanoma and the RENCA renal cell cancer model, CPI-444 induced antitumor immune responses, and suppressed tumor growth as a single agent; it also augmented the efficacy of anti-PD-1/PD-L1 and anti-CTLA-4 agents [19]. CPI-444 was also reported to decrease the expression of multiple checkpoint pathways and to improve T cell infiltration and effector functions in MC-38, CT-26, and B16OVA tumor models [37]. In February 2015, CPI-444 was licensed by Corvus Pharmaceuticals for further development of therapeutic applications.

A clinical study of CPI-444 to evaluate its pharmacokinetics has been completed (NCT03237988), and a phase I/Ib study evaluating the safety and clinical activity of CPI-444 alone and in combination with anti-PD-1 atezolizumab in patients with advanced solid tumors was initiated in 2016 (NCT02655822). In April 2018, Corvus sponsored a study of CPI-006, an anti-CD73 mAb, alone and in combination with CPI-444 and pembrolizumab for patients with advanced cancers (NCT03454451). A phase Ib study of CPI-444 as a single agent and in combination with the anti-CD38 antibody daratumumab in relapsed or refractory multiple myeloma was initiated in February 2020 (NCT04280328). Results from these trials should be available in the near future.

### 3.8. AB-928

AB-928 is an A_2A_AR and A_2B_AR dual antagonist discovered by Arcus Biosciences, which inhibits A_2A_AR and A_2B_AR with similar potencies (K_i_ = 1.5 and 2.0 nM, respectively) and with 42- and 326-fold selectivity against A_1_AR and A_3_AR, respectively (Table 1) [42]. Given that both A_2A_AR and A_2B_AR play important roles in the immunosuppression by adenosine, dual antagonists may have better anticancer effects [4]. In a preclinical model of AT-3-OVA-bearing mice, AB-928 combined with chemotherapy by doxorubicin or oxaliplatin resulted in a significant reduction in the tumor growth rate when compared to chemotherapy alone [65]. In healthy volunteers, AB-928 was well tolerated up to the highest dose tested (200 mg once daily) and did not affect any physiologic parameters that are potentially sensitive to adenosine inhibition [66]. Researchers found that plasma levels of over 1 μM of the compound were associated with over 90% adenosine receptor inhibition [66]. These data resulted in further clinical development of oral AB-928 in cancer patients [66].

A number of clinical trials have been initiated to study the anticancer effects of AB-928 in combination with other agents. For example, a phase I study to evaluate the safety and tolerability of AB-928 in combination with the anti-PD-1 antibody zimberelimab in patients with advanced malignancies was started in August 2018 (NCT03629756), and a phase I study to evaluate the clinical efficacy of AB-928 combined with the drug combination “mFOLFOX” in participants with advanced metastatic gastroesophageal cancer was started in October 2018 (NCT03720678). In January 2020, a phase II study to evaluate AB-928 in combination with the anti-PD-1 zimberelimab or with AB-154 (a humanized mAb targeting human T cell immune-receptor with Ig and ITIM domains (TIGIT)) in participants with PD-L1-positive NSCLC, was started (NCT04262856). In June 2020, a phase Ib/II study to evaluate the antitumor activity and safety of an AB-928-based combination therapy in participants with metastatic castrate resistant prostate cancer in which AB-928 was combined with zimberelimab, enzalutamide, docetaxel, and the anti-CD73 antibody AB-680 was started (NCT04381832). Data from these trials should reveal the efficacy of A_2A_AR and A_2B_AR dual antagonists in cancer immunotherapy.

### 3.9. EOS-100850

EOS-100850 is an A_2A_AR antagonist reported by iTeos Therapeutics for which the chemical structure has not been disclosed as yet. Based on a patent publication by iTeos [67], EOS-100850 may contain a novel core structure (5-aminothiazolo[5,4-e][1,2,4]triazolo[1,5-c]pyrimidin-2(3H)-one), which would be similar to the tricyclic structure of A2AAR antagonists, such as SCH-58261 and MK-3814. Little has been published regarding the preclinical profiling of this compound. A phase I/Ib first-in-human study of EOS-100850 in patients with advanced solid tumors was started in January 2019 (NCT03873883). Clinical studies of EOS-100850 in combination with other agents in patients with melanoma, prostate cancer, and triple-negative breast cancer have also been planned [68].

## 4. Binding Modes of Antagonists in Complexes with A_2A_AR

For four of the antagonists presented in Figure 2 and Table 1, crystal structures of complexes with A_2A_AR are available. These structures greatly contribute to the understanding of the binding modes of A_2A_AR ligands and, therefore, help with the design of novel lead compounds for development of drugs that target A_2A_AR. This section surveys the available structures.

**Figure 3:**
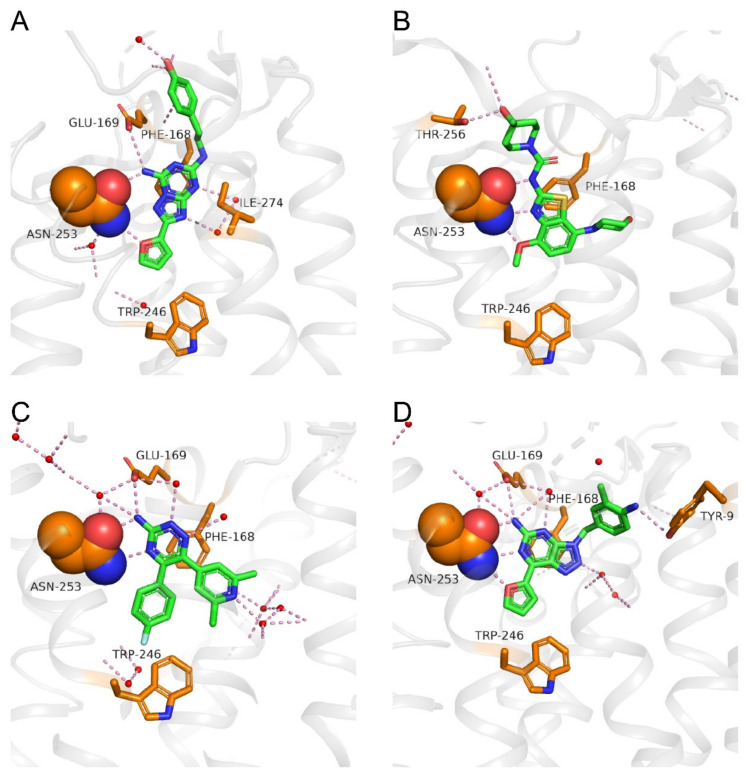
Binding modes of A_2A_AR with different antagonists. (**A**) ZM-241385; the key amino acid residues: Asn253^6.55^, Glu169^5.30^, Phe168^5.29^, Trp246^6.48^, and Ile274^7.39^ are identified (PDB ID: 3EML). (**B**) SYN-115; the key amino acid residues: Asn253^6.55^, Thr256^6.58^, Phe168^5.29^, and Trp246^6.48^ are identified (PDB ID: 5OLO). (**C**) AZD-4635; the key amino acid residues: Asn253^6.55^, Glu169^5.30^, Phe168^5.29^, and Trp246^6.48^ are identified (PDB ID: 6GT3). (**D**) V-2006; the key amino acid residues: Asn253^6.55^, Glu169^5.30^, Tyr9^1.35^, Phe168^5.29^, and Trp246^6.48^ are identified (PDB ID: 5OLH). The A_2A_AR back bone is colored gray, and the amino acid side chains that interact with the ligands are shown as sticks and colored by element (carbon, yellow; nitrogen, blue; oxygen, red; sulfur, yellow; the side chain of Asn253^6.55^ is shown in a space filling presentation). The antagonists are shown as sticks and colored by element (carbon, green; nitrogen, blue; oxygen, red; sulfur, yellow). Polar contacts are presented as dashed lines, and water molecules are shown as red spheres.

A crystal structure of ZM-241385 bound to A_2A_AR solved in 2008 (PDB: 3EML) [20] showed the inactive conformation of the receptor (Figure 3A). ZM-241385 binds to the receptor in an extended conformation, with the furan head inserting deep into the binding pocket and the phenol tail located at the entry of the orthosteric binding site [20]. The ligand-binding pocket is bottomed by Trp246^6.48^, and ZM-241385 is anchored by an aromatic stacking interaction with Phe168^5.29^. The furan ring forms a hydrogen bond with the side chain NH_2_ group of Asn253^6.55^, and the NH_2_ group on the bicyclic triazolotriazine core of ZM-241385 forms hydrogen bonds with the side chain C=O groups of Asn253^6.55^ and Glu169^5.30^. The tail phenol group of ZM-241385 forms a hydrogen bond with a water molecule at the pocket entry. In a different ZM-241385-bound A_2A_AR structure solved in 2011, a salt bridge between His264^ECL3^ and Glu169 is broken (not shown in Figure 3A), and the tail phenol group points to a side pocket instead of the pocket entry (PDB: 3PWH) [69].

A crystal structure of SYN-115 bound to A_2A_AR reported in 2018 (PDB: 5OLO) [70] shows that SYN-115 fits into a similar pocket to that occupied by ZM-241385 (Figure 3A,B). There is an aromatic stacking interaction between the benzothiazole core of SYN-115 and residue Phe168^5.29^. Three hydrogen bonds could be seen between the ligand and the side chain of Asn253^6.55^ in which the oxygen of the methoxy group and the nitrogen of the thiazole ring interact with the side chain NH_2_ group of Asn253^6.55^ and act as hydrogen bond acceptors, while the NH of SYN-115 forms a hydrogen bond with the side chain C=O group of Asn253^6.55^. Similar to ZM-241385, the hydroxyl tail of SYN-115 points toward the pocket entry, where it forms a hydrogen bond with Thr256^6.58^.

Crystal structures of AZD-4635 analogs (3-amino-1,2,4-triazine compounds) bound to the A_2A_AR reported in 2012 [71], and a more recently presented higher resolution structure of AZD-4635-bound A_2A_AR (PDB: 6GT3) show that AZD-4635 lies deep in the binding pocket and occupies a region next to a cluster of “unhappy” waters (Figure 3C). Unlike ZM-241385 or SYN-115, AZD-4635 does not have a “tail” group that would reach out to the pocket entry. Hydrogen bonds are observed between the triazine nitrogen at position 4 and the side chain NH_2_ group of Asn253^6.55^ and between the NH_2_ group on the triazine ring and the side chain C=O groups of Asn253^6.55^ and Glu169^5.30^.

A crystal structure of compound V-2006 bound to A_2A_AR (PDB: 5OLH) [70] shows that the bicyclic core and the furan substituent of V-2006 share very similar interactions to those of ZM-241385, namely the aromatic stacking with Phe168^5.29^ and the hydrogen bonds with Asn253^6.55^ and Glu169^5.30^ (Figure 3D). Unlike ZM-241385, the 2-methylaniline group of V-2006 does not point to the pocket entry but fits into a side pocket, where it interacts with Tyr9^1.35^.

Overall, although the chemical structures of the four A_2A_AR antagonists (Figure 2) are quite different, they are buried in the orthosteric pocket of A_2A_AR, where they show many common interactions with key residues in the pocket (Figure 3). The basal region of the orthosteric site is delimited by Trp246^6.48^, which engages in Van der Waals contacts to aromatic substituents, such as furan (ZM-241385 and V-2006) and benzene (AZD-4635). The aromatic stacking interaction with Phe168^5.29^ and the hydrogen bonds with Asn253^6.55^ are common interactions for all four A_2A_AR antagonists. When a –NH_2_ group is attached to the bicyclic or tricyclic core of A_2A_AR antagonists (Figure 2), a hydrogen bond with Glu169^5.30^ is often seen. Additional studies of crystal structures of A_2A_AR complexes with different ligands and their binding kinetics [23,52,72,73,74] also contribute to the foundation for the discovery of new chemotypes of A_2A_AR antagonists, either through virtual screening approaches or structure-based drug design.

## 5. Conclusions and Future Perspectives

In the tumor microenvironment, adenosine suppresses the antitumor activity of effector T cells and other cytotoxic immune cells mainly through its action on A_2A_AR. Blocking A_2A_AR thus has the potential to markedly enhance antitumor immunity. Preclinical in vivo animal studies have demonstrated efficacy of A_2A_AR antagonists in tumor immunotherapy, providing incentives to develop A_2A_AR antagonists for use in immuno-oncology.

The complex pathogenesis of all cancerous growths makes it challenging to achieve efficacious and lasting therapeutic effects for single-drug therapies, mainly because of imminent drug resistance. To obtain more effective antitumor effects, drug combinations are commonly applied. This is also true for A_2A_AR antagonists. In most preclinical models, A_2A_AR antagonists show only mild tumor-suppressing effects as stand-alone agents, while significantly better results could be achieved when A_2A_AR antagonists are used in combination with other antitumor agents. In clinical trials, A_2A_AR antagonists are most often tested in combination with other check-point inhibitors (in most cases anti-PD-1/PD-L1 or anti-CTLA-4 antibodies) as well as with chemotherapies. Ongoing studies will reveal novel combination regimens, improved dosing and timing of interventions in different tumor types and patient subpopulations.

Based on the prominent role of A_2A_AR in immuno-oncology, new antagonist chemotypes are being developed based on structural information on ligand binding modes as well as reported structure–activity relationships. Because of the high adenosine concentrations in the TME, high binding affinity is necessary for A_2A_AR antagonist drug candidates. Target selectivity is of course also very important, especially versus A_1_AR and A_3_AR because of their opposite downstream effects on the regulation of cAMP levels. Selectivity versus A_2B_AR is a quite different issue, as illustrated by the fact that dual A_2A_AR and A_2B_AR antagonists are also being developed. Finally, considering that otherwise potentially promising A_2A_AR antagonists had to be abandoned in drug-discovery projects because of poor physicochemical and pharmacokinetic properties, multi-parametric optimization of lead compounds is yet another avenue to be further explored in the development of clinically useful A_2A_AR antagonists.

## Figures and Tables

**Table 1:** A_2A_AR antagonists and their affinity for adenosine receptors.

Compound	K_i_ (nM)				References
	A_1_AR	A_2A_AR	A_2B_AR	A_3_AR	
KW-6002	9600	12	1800	>3000	[44]
ZM-241385	255	0.8	50	>10,000	[45,46]
SCH-58261	287	0.6	5011	>10,000	[47]
MK-3814	>1000	1.1	>1700	>1000	[48]
PBF-509	2500	12	1000	5000	[18]
SYN-115	1350	5	700	1570	[49]
AZD-4635	160	1.7	64	>10,000	[50]
V-2006	68	1.3	63	1005	[51]
CPI-444	192	3.54	1528	2455	[19]
AB-928	64	1.5	2.0	489	[42]
